# Can Intermittent Hypoxic Conditioning Enhance the Benefits of Standard Long COVID‐19 Rehabilitation?

**DOI:** 10.1002/jcsm.13769

**Published:** 2025-03-04

**Authors:** Tim Kambič, Tadej Debevec, Mitja Lainscak

**Affiliations:** ^1^ Faculty of Sport University of Ljubljana Ljubljana Slovenia; ^2^ Department of Medical BioSciences, Exercise Physiology Research Group Radboud University Medical Centre Nijmegen the Netherlands; ^3^ Department of Automatics, Biocybernetics and Robotics Jožef Stefan Institute Ljubljana Slovenia; ^4^ Faculty of Medicine University of Ljubljana Ljubljana Slovenia; ^5^ Division of Cardiology General Hospital Murska Sobota Murska Sobota Slovenia

**Keywords:** exercise training, hypoxia exposure, post COVID‐19, pulmonary rehabilitation, SARS‐CoV‐2

Since the outbreak of the Coronavirus‐19 (COVID‐19) pandemic in December 2019, nearly 705 million people got infected with severe acute respiratory syndrome coronavirus 2 (SARS‐CoV‐2) and more than 7 million people died [1]. Following recovery from acute COVID‐19 infection, many individuals continue to suffer from long‐term sequelae of SARS‐CoV‐2 infection (≥ 3 months) defined as long COVID‐19 syndrome or post COVID condition [2, 3, 4]. Unsurprisingly, incidence of long COVID‐19 syndrome is the highest among hospitalised unvaccinated individuals (50%–85%) and the lowest among vaccinated people (8–12%) and in most cases persisted more than 1 year [2]. The greater risk and most severe symptoms of long COVID‐19 were manifested in females aged 35–50 years, patients with comorbidities (type 2 diabetes, allergies, lung diseases, heart failure, chronic kidney disease and obesity) and in unvaccinated individuals [2, 3, 4, 5]. Sarcopenia is highly prevalent in patients with long COVID‐19, particularly after longer hospitalisation [6].

Long COVID‐19 is a heterogeneous, systemic and multiorgan syndrome that has been associated with > 200 different symptoms. The accumulation of SARS‐CoV‐2 in the tissues, dysregulated immune response, inflammation and endothelial dysfunction induce damage to pulmonary (lung fibrosis and alveolar epithelium injury), cardiovascular (peri‐myocarditis, arterial inflammation, micro‐thrombosis and coagulopathy), skeletal muscle (muscle fibre atrophy and disrupted mitochondrial function) [7, 8], neurological (postural orthostatic tachycardia and dysautonomia) and autoimmune (onset of autoimmune diseases) systems (Figure 1) [2, 3, 4, 9]. These impairments are primarily manifested as fatigue, cough, shortness of breath at rest and after exercise, exercise intolerance, chest pain, joint and muscle pain, cognitive impairments (dizziness, brain fog) and sleep disorders, along with other less frequent symptoms [2, 3, 4, 7, 9, 10].

**FIGURE 1:**
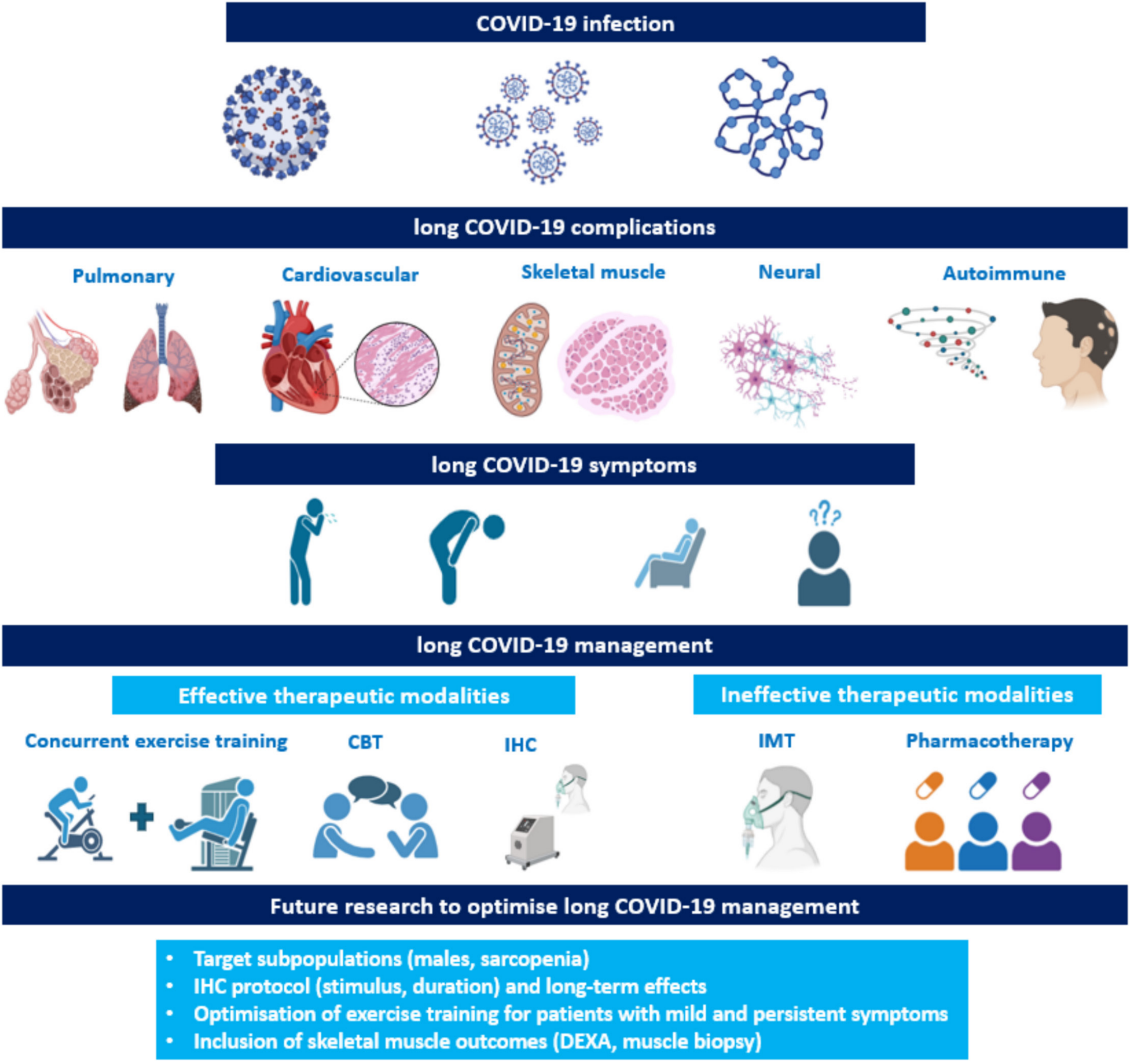
Frontiers in management of long COVID‐19. CBT, cognitive behavioural training; COVID‐19, coronavirus‐19; DEXA, dual‐energy X‐ray absorptiometry; IHC, intermittent hypoxic conditioning; IMT, inspiratory muscle training.

Multisystem involvement makes long COVID‐19 management challenging. To date, only cognitive and exercise training interventions alone or combined have shown promise of improvement in cognitive, pulmonary and physical function over usual care; a plethora of other interventions, including drugs, food supplementation and neurological approaches, did not provide benefits (Figure 1) [11, 12]. With pulmonary symptoms at a centre stage, several exercise training modalities for patients with long‐COVID were effectively translated from standard pulmonary rehabilitation [13], while the inspiratory muscle training alone demonstrated no added benefit. Therefore, the search for safe and effective pulmonary or systemic intervention that would additionally restore pulmonary function continues.

Over the recent years, the manipulations of ambient oxygen (O_2_) (e.g., hypoxic and hyperoxic conditioning) have emerged as a therapeutic strategy from sports performance settings, which lead to development of different hypoxic interventions, including intermittent hypoxia conditioning (IHC; e.g., short daily repeated bouts of breathing moderate hypoxia [F_IO2_ usually 9–15%] interspersed with intervals breathing ambient air) [14, 15, 16]. The application of IHC can provoke improvements in many physiological and functional systems (e.g., cardiopulmonary function, exercise performance, glucose metabolism and weight management) in both athletes and chronic disease patients [14, 15, 16, 17, 18]. In patients with cardiovascular disease, resting IHC reduced resting heart rate, systolic and diastolic blood pressure [17]. In addition, early studies from the Soviet Union also suggested potential improvements in myocardial function, blood pressure and sympathetic nervous system regulation [16].

As previous clinical studies in cardiometabolic patients [14, 16, 17, 18] suggested benefits of IHC for use in rehabilitation of patients with long COVID‐19, Doehner et al. sought to determine whether such benefits can be translated to rehabilitation of patients with long COVID‐19 in a pilot trial recently published in the *Journal* [19]. The study included 145 overweight, middle‐aged patients with long COVID‐19 and coexisting arterial hypertension that were allocated to 5 weeks of either combination of IHC with extensive, multidisciplinary rehabilitation program (IHC + rehab, *n* = 70) or to extensive, multidisciplinary rehabilitation programme alone (rehab, *n* = 75). The IHC intervention was performed in three weekly sessions consisting of rather standardised six to eight intervals of 3–5 min of breathing a hypoxic air mixture (10%–12% of O_2_) interspersed with 3–5 min of breathing a slightly hyperoxic air mixture (30%–35% of O_2_). After 45 min of IHC, patients continued with multimodal rehabilitation consisting of *physical therapy* (inspiratory muscle training, electrotherapy, hydrotherapy, etc), *exercise training* (progressive aerobic, resistance, balance and coordination training), *occupational training* (cognitive and motor training for improving activities of daily life) and *interdisciplinary educational counselling* with *psychosocial support* focused on improving self‐management of long COVID‐19 disease [19]. The primary study outcome was the change in (sub)maximal endurance (6‐min walk test distance) along with integrative assessment of strength (hand grip strength, nine‐hole peg test, timed up‐and‐go test and functional ambulatory capacity), pulmonary capacity, symptoms of dyspnoea, health‐related quality of life, blood pressure and biomarkers of inflammation, glucose metabolism and kidney function [19]. The combination of IHC and multidisciplinary rehabilitation appeared safe, well‐tolerated and showed a superior effect on improving 6 ‐min walk test distance (+59 m), stair climbing time (−1.4 s), health‐related quality of life (European Quality of Life Five Dimensions Questionnaire analogue scale: +28.4 points) and on long COVID‐19 symptoms (Median COVID‐19 Recovery Score: −10.2 points) compared with multidisciplinary rehabilitation alone.

While Doehner et al. [19] should be applauded for taking the first step in exploring the potential of IHC as added tool to standard, mainly exercise‐based rehabilitation of patients with long COVID‐19, there remain some important considerations for advancing our understanding and applicability of this approach. First, the study findings should be tested in future well‐powered randomised, controlled trials, with special emphasis on the appropriate group allocation, which represents one of the limitations in the present study and might partially underscored primary outcome (6‐min walk test). Future work should also aim to balance biological sex in the sample, as the present study enrolled mostly female patients likely due to higher prevalence of the syndrome compared to males [2, 3, 19]. Second, future studies should put special emphasis on improving prescription and progression of exercise training, particularly resistance training [20, 21], to counterbalance the cardiopulmonary and skeletal muscle impairments associated with long COVID‐19 sequalae [7], which may accelerate sarcopenia in these patient group [2, 6]. Optimised resistance training should be complemented with the inclusion of advanced measurement of body composition (e.g., dual‐energy X‐ray absorptiometry) and skeletal muscle histology and metabolism (e.g., biopsies on *m. vastus lateralis*) to gain novel insights into the changes of muscle quality and quantity (Figure 1). Nevertheless, Doehner et al. [19] provide a strong foundation to optimise future exercise training interventions, as solely multidisciplinary rehabilitation approach demonstrated clinical meaningful improvements in 6‐min walk test. Thirdly, the benefits of current somewhat standardised IHC protocol combined with pulmonary rehabilitation [13] can be enhanced with further IHC protocol adjustments (changes in hypoxia/hyperoxia ratio, extended exposure per set and within single session) [14], especially for long COVID‐19 patient with severe exercise participation‐limiting symptoms. Lastly, the benefits of combined IHC with standard pulmonary rehabilitation [13] should be tested in a longer intervention (8–12 weeks) with extended follow‐up (≥ 1 year), to investigate whether IHC effects are maintained over longer period of time or whether long COVID‐19 symptoms can present again [2].

The application of IHC, as suggested by Doehner et al. [19], should undoubtedly be considered in rehabilitation of patients with long COVID‐19 syndrome and can be effectively combined within the current scheme of standard pulmonary rehabilitation [13]. After extensive clinical assessment [13, 19], the IHC can be introduced in the early phases of long COVID‐19 rehabilitation, preferably immediately after COVID‐19 infection hospitalisation for patients with severe symptoms of breathlessness and exercise intolerance. Therefore, in early phases of rehabilitation (1–4 weeks), IHC can be added to inspiratory muscle training, low‐to‐moderate intensity continuous aerobic training (40%–60% of peak O_2_ consumption, 45–60 min) and resistance training (3–5 sets of 15–20 repetitions at 40%–60% of maximal muscle strength), balance training and other cognitive and physical therapy interventions [11, 13]. In the later phases of long COVID‐19 rehabilitation (5–12 weeks), the intensity of exercise training can be progressively increased (aerobic training: 60%–80% of peak O_2_ consumption; resistance training: 70%–80% of muscle strength) [13, 21], while the use of IHC and balance training should be predominantly applied for patients with persistent moderate‐to‐severe symptoms of breathlessness, exercise intolerance and dizziness and vertigo, respectively. Such individualised approach may further optimise the intensive multidisciplinary rehabilitation of long COVID‐19 and consequently help reduce the long‐term burden of the syndrome [2, 3, 10, 21].

In summary, the future of multimodal long COVID‐19 rehabilitation consisting of concurrent exercise training [21] and behavioural interventions [11] combined with effective IHC looks bright. Prospective work is nevertheless, warranted, to provide further insights into the question whether the short‐term effects of IHC timely presented by Doehner et al. [19] can be maintained/enhanced by using a longer, individually tailored interventions for the most vulnerable patients' populations with persistent long COVID‐19 syndromes (patients with pre‐existing sarcopenia, after longer hospitalisation and/or with multiple metabolic and cardiopulmonary comorbidities).

## Conflicts of Interest

The authors declare no conflicts of interest.
